# Prediction of Hematopoietic Stem Cell Transplantation Related Mortality- Lessons Learned from the *In-Silico* Approach: A European Society for Blood and Marrow Transplantation Acute Leukemia Working Party Data Mining Study

**DOI:** 10.1371/journal.pone.0150637

**Published:** 2016-03-04

**Authors:** Roni Shouval, Myriam Labopin, Ron Unger, Sebastian Giebel, Fabio Ciceri, Christoph Schmid, Jordi Esteve, Frederic Baron, Norbert Claude Gorin, Bipin Savani, Avichai Shimoni, Mohamad Mohty, Arnon Nagler

**Affiliations:** 1 Division of Hematology and Bone Marrow Transplantation, The Chaim Sheba Medical Center, Tel-Hashomer, Ramat-Gan, Israel; 2 Internal medicine "F" Department and the 2013 Pinchas Borenstein Talpiot Medical Leadership Program, The Chaim Sheba Medical Center, Tel-Hashomer, Ramat-Gan, Israel; 3 The Mina and Everard Goodman Faculty of Life Sciences, Bar-Ilan University, Ramat-Gan, Israel; 4 EBMT Paris Office, Hospital Saint Antoine, Paris, France; 5 Sorbonne Universités, UPMC Univ Paris 06, UMR_S 938, CDR Saint-Antoine, F-75012, Paris, France; 6 INSERM, UMR_S 938, CDR Saint-Antoine, F-75012, Paris, France; 7 AP-HP, Hématologie Clinique et Thérapie Cellulaire, Hôpital Saint-Antoine, Paris, France; 8 Department of Bone Marrow Transplantation and Oncohematology, Maria Sklodowska-Curie Memorial Cancer Center and Institute of Oncology, Gliwice Branch, Gliwice, Poland; 9 Hematology and BMT Unit, San Raffaele Scientific Institute, Milan, Italy; 10 Department of Hematology and Oncology, Klinikum Augsburg, Ludwig-Maximilians-University, Munich, Germany; 11 Hematology Department, IDIBAPS, Hospital Clínic, Barcelona, Spain; 12 Hematology & GIGA research, University of Liège, Liège, Belgium; 13 Hematology & Stem Cell Transplantation Section, Vanderbilt University Medical Center, Nashville, Tennessee, United States of America; European Institute of Oncology, ITALY

## Abstract

Models for prediction of allogeneic hematopoietic stem transplantation (HSCT) related mortality partially account for transplant risk. Improving predictive accuracy requires understating of prediction limiting factors, such as the statistical methodology used, number and quality of features collected, or simply the population size. Using an *in-silico* approach (i.e., iterative computerized simulations), based on machine learning (ML) algorithms, we set out to analyze these factors. A cohort of 25,923 adult acute leukemia patients from the European Society for Blood and Marrow Transplantation (EBMT) registry was analyzed. Predictive objective was non-relapse mortality (NRM) 100 days following HSCT. Thousands of prediction models were developed under varying conditions: increasing sample size, specific subpopulations and an increasing number of variables, which were selected and ranked by separate feature selection algorithms. Depending on the algorithm, predictive performance plateaued on a population size of 6,611–8,814 patients, reaching a maximal area under the receiver operator characteristic curve (AUC) of 0.67. AUCs’ of models developed on specific subpopulation ranged from 0.59 to 0.67 for patients in second complete remission and receiving reduced intensity conditioning, respectively. Only 3–5 variables were necessary to achieve near maximal AUCs. The top 3 ranking variables, shared by all algorithms were disease stage, donor type, and conditioning regimen. Our findings empirically demonstrate that with regards to NRM prediction, few variables “carry the weight” and that traditional HSCT data has been “worn out”. “Breaking through” the predictive boundaries will likely require additional types of inputs.

## Introduction

Allogeneic hematopoietic stem transplantation (HSCT) is a potentially curative procedure for selected patients with hematological malignancies. Transplant associated morbidity and mortality remains substantial, making the decision of whom, how and when to transplant, of great importance [1].

The European Group for Blood and Marrow Transplantation (EBMT) score, initially developed for prediction of allogeneic HSCT outcomes in chronic myeloid leukemia, and later validated for other diagnoses, has pioneered the field of prognostic modeling in HSCT [2, 3]. Since its release, almost two decades ago, additional scores have also been developed. These have been validated, but do not fully account for transplantation risk in acute leukemia [4–9].

Performance limiting factors of HSCT prediction models might be attributed to inherent procedural uncertainty, the statistical methodology used, or the number and quality of features collected. Using an *in-silico* approach (i.e., iterative computerized simulations), based on machine learning (ML) algorithms, we set out to explore these factors in order to improve future acute leukemia HSCT outcome prediction models.

ML is a field in artificial intelligence. The underlying paradigm does not start with a pre-defined model; rather it lets the data create the model by detecting underlying patterns. Thus, this approach avoids pre-assumptions regarding model types and variable interactions, and may offer additional knowledge, which has eluded detection by standard statistical methods. ML algorithms, have been applied in various "big data" scenarios such as financial markets, complex physical systems, marketing, advertising, robotics, meteorology, biology and more. They are tools in the data mining approach for knowledge discovery in large datasets [10, 11]. Recently, we have developed the EBMT- Alternating Decision Tree (ADT) ML based prediction model for mortality at 100 days following allogeneic HSCT in acute leukemia [9, 12]. Hence, demonstrating feasibility of the data mining approach in HSCT.

## Methods

### Study population

This was a retrospective, data mining, supervised learning study, based on data reported to the Acute Leukemia Working Party (ALWP) registry of the EBMT. The EBMT is a voluntary group of more than 500 centers, required to report all consecutive HSCT and follow-ups annually in a standardized manner. The study was approved by the ALWP board. Written informed consent was given by participants for their clinical records to be used in EBMT retrospective studies.

Inclusion criteria encompassed first allogeneic transplants from HLA matched sibling and unrelated donors (> = 8/10), performed from 2005 to 2013, using peripheral blood stem cells or bone marrow as cell source, on adults (age > = 18 years) diagnosed with de-novo acute leukemia. Haploidentical and cord blood transplants were not included.

A total of 26,266 patients from 326 European centers were initially analyzed. Patients lost from follow-up before day 100 post HSCT were discarded from analysis (n = 343, 1.3%). Twenty two variables describing recipient, donor, and procedural characteristics were considered.Variables were defined according to EBMT criteria (Table 1 and Appendix A in S1 File) [13].

**Table 1:** Patient Characteristics. Interquartile range (IQR), Body mass index (BMI), Recipient (R), Donor (D), Cytomegalovirus (CMV), Acute lymphoblastic leukemia (ALL), Total body irradiation (TBI), Graft versus host disease (GVHD), Antithymocyte globulin (ATG), Peripheral blood (PB), Bone marrow (BM)

	Value	N	Missing, n
Median year (IQR)	2009 (2007–2011)	25923	0
Median recipient age (IQR)	45 (33–56)	25923	0
Median BMI (IQR)	24 (22–27)	9350	16573
Median days between diagnosis and HSCT (IQR)	191 (138–363)	25914	9
Median donor's age (IQR)	38.8 (29–48)	10027	15896
Recipient gender		25872	51
	Male	14228 (55.0%)	
	Female	11644 (45.0%)	
Recipient CMV serostatus		22855	3068
	-	7788 (34.1%)	
	+	15067 (65.9%)	
Karnofsky at transplant		24369	1554
	> = 80	22966 (94.2%)	
	<80	1403 (5.8%)	
Comorbidity score merged		2469	23454
	0	403 (16.3%)	
	1	747 (30.3%)	
	2	457 (18.5%)	
	3	419 (17.0%)	
	> = 4	443 (17.9%)	
Diagnosis		25923	0
	AML	18610 (71.8%)	
	ALL	7313 (28.2%)	
Cytogenetics risk		13430	12493
	Standard	10080 (75.1%)	
	Poor	3350 (24.9%)	
Disease stage		25923	0
	CR1	16201 (62.5%)	
	CR2	4909 (18.9%)	
	Advanced	4813 (18.6%)	
Previous autograft		25923	0
	-	25235 (97.3%)	
	+	688 (2.7%)	
Donor gender		25357	566
	Male	15712 (62.0%)	
	Female	9645 (38.0%)	
Donor CMV serostatus		22726	3197
	-	10927 (48.1%)	
	+	11799 (51.9%)	
D-R sex combination		25318	605
	Male D to male R	9153 (36.2%)	
	Female D to female R	4863 (19.2%)	
	Male D to female R	6528 (25.8%)	
	Female D to male R	4774 (18.9%)	
D-R CMV serostatus combination		22395	3528
	D-CMV–/R-CMV–	5572 (24.9%)	
	D-CMV+/R-CMV–or D-CMV–/R-CMV+	8917 (39.8%)	
	D-CMV+/R-CMV+	7906 (35.3%)	
Donor type		25923	0
	HLA matched unrelated donor	13585 (52.4%)	
	HLA identical sibling	12338 (47.6%)	
HLA match degree		9090	16833
	10/10	6519 (71.7%)	
	9/10	2068 (22.8%)	
	<9/10	503 (5.5%)	
Source of stem cells		25923	0
	BM	4109 (15.9%)	
	PB or BM+PB	21814 (84.1%)	
Conditioning		25420	503
	MAC	16836 (66.2%)	
	RIC	8584 (33.8%)	
TBI		25742	181
	No	15042 (58.4%)	
	Yes	10700 (41.6%)	
GVHD prevention		23228	2695
	Ex-vivo T cell depletion	800 (3.4%)	
	In-vivo T cell depletion	9825 (42.3%)	
	No T cell depletion	12603 (54.3%)	
Relapse at day 100		25923	0
	-	23384 (90.2%)	
	+	2539 (9.8%)	
Non relapse related mortality at day 100		25923	0
	-	23536 (90.8%)	
	+	2387 (9.2%)	
Overall mortality at day 100		25923	0
	-	22643 (87.3%)	
	+	3280 (12.7%)	

### Study objectives

Study objectives included development of multiple prediction models for NRM 100 days post allogeneic HSCT, while estimating effects of the algorithm type, population size, specific subpopulations and number of variable incorporated, on the models' predictive performance. Day 100 NRM was defined as death without previous relapse/progression before day 100.

### Study design

**Fig 1:**
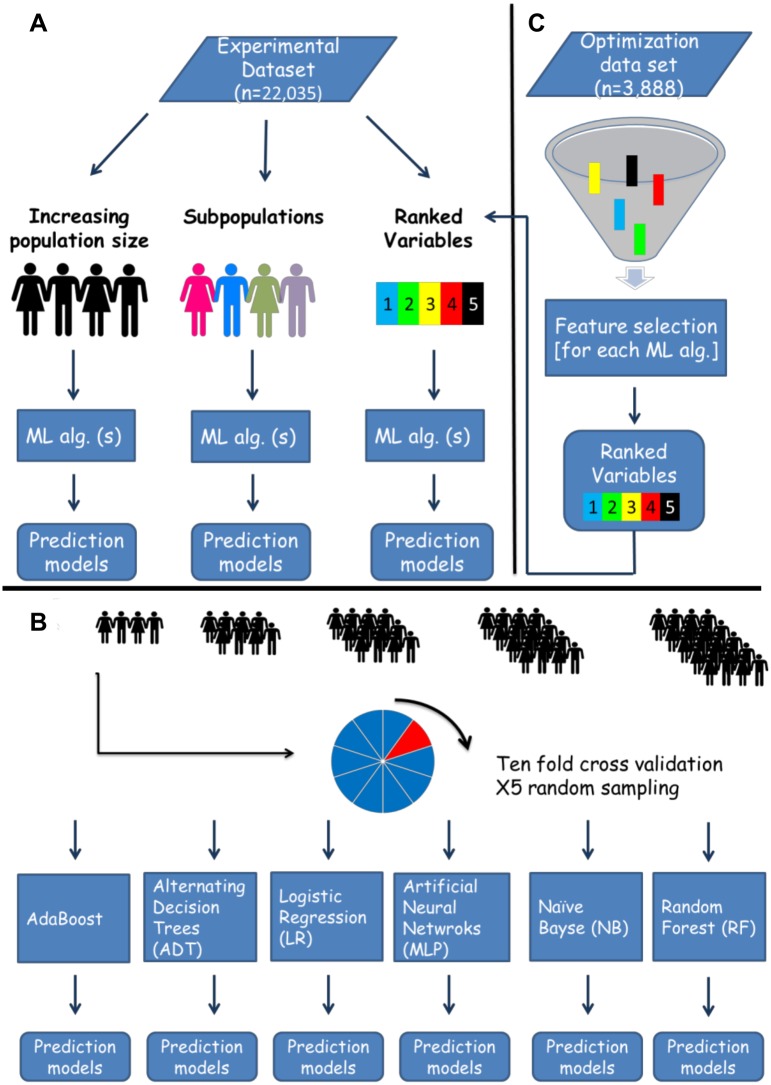
*In-silico* predictive modeling- experimental design. The original dataset was randomly split into an optimization and experimental datasets. The former was used for tuning of machine learning algorithms and feature selection. **A**. Several experiments were run on the experimental dataset, testing the effects of population size, specific subpopulations and number of variables included on predictive performance. **B**. A detailed explanation of the increasing population size experiment displayed in panel A. Patients were randomly sampled from the experimental dataset, creating samples with an expending size, which were later introduced to six machine learning algorithms. For each sample a prediction model for day 100 NRM was developed, and performance was measured through the area under the receiver operating curve (AUC). Models were trained and tested with 10 fold cross validation. The sampling process was repeated 5 times. **C**. For estimation of variable importance (ranked variables experiment in panel A) and the number of variables necessary for optimal prediction of day 100 NRM, we ran a feature selection algorithm on the optimization set. Variables were ranked according to their predictive contribution to each algorithm. The next step involved serial introduction of the variables, according to their importance to six machine learning algorithms which were applied on the experimental dataset. In each iteration a prediction model for day 100 NRM was trained and test with 10 fold cross validation. For instance in the first iteration the top ranking variable was introduced, in the second the top 2 variables and so on until all 23 variables were used. Performance was estimated according to the AUC. Machine learning (ML), Algorithm (Alg).

Prediction models for day 100 NRM were developed using six ML algorithms (WEKA v. 3-7-11, New-Zealand). Through an *in-silico* approach, algorithms were iteratively exposed to an increasing population size, varying sub-populations, or an increasing number of ranked variables, selected by a separate feature selection algorithm (Fig 1). For each iteration, a prediction model was trained and tested through 10 fold cross-validation. This process was repeated 5 times, each time randomly sampling the experimental dataset (see below). Performance was evaluated according to the area under receiver operator characteristic curve (AUC) [14, 15].

Tuning of the algorithms parameters (Table A in S1 File) and the feature selection process, explained below, were conducted on an optimization dataset (n = 3,888, 15%), whereas the development of the various models of day 100 NRM prediction were done on the experimental dataset (n = 22,035, 85%). Samples were randomly allocated to each dataset from the original dataset.

### Machine learning Algorithms

Six popular, supervised classification ML algorithms were selected (Appendix B in S1 File). Naïve bayse (NB), alternating decision trees (ADT) and logistic regression (LR) produce models with interpretable structures, whereas multilayer perceptron (MLP), random forest (RF) and AdaBoost are "black box" models, where the function connecting the predictor variables with response is opaque to the user [16–22].

### Feature selection

Feature selection is the process of ranking variables and identifying irrelevant and redundant information. The reduction of dimensionality presents a number of benefits, such as enabling algorithms to operate faster and more effectively, improving classification accuracy, improving data visualization, and enhancing understanding of the derived classification models [23]. Using a classifier based feature selection algorithm, which was applied on the optimization dataset for each of the 6 previously described ML classification algorithms, variables were ranked according to their importance for prediction of day 100 NRM (Appendix C in S1 File).

## Results

### Patient characteristics

Characteristics of 25,923 analyzed patients are listed in Table 1. The majority had Acute Myeloid Leukemia (AML) (71.8%), were in first complete remission (CR1) (62.5%) and received myeloablative conditioning (MAC) (66.2%). Grafts from matched sibling donors were used in 47.6% of patients. Graft source was mainly peripheral blood (84.1%). NRM and overall mortality prevalence at day 100, were 9.2% (n = 2,387) and 12.7% (n = 3,280) respectively. Whereas 9.8% (n = 2,539) of patients relapsed before 100 days. They were consequently considered as no NRM at day100. The parameter optimization and experimental datasets were similar in terms of baseline characteristics (Table B in S1 File).

### Sample size effect on prediction

**Fig 2:**
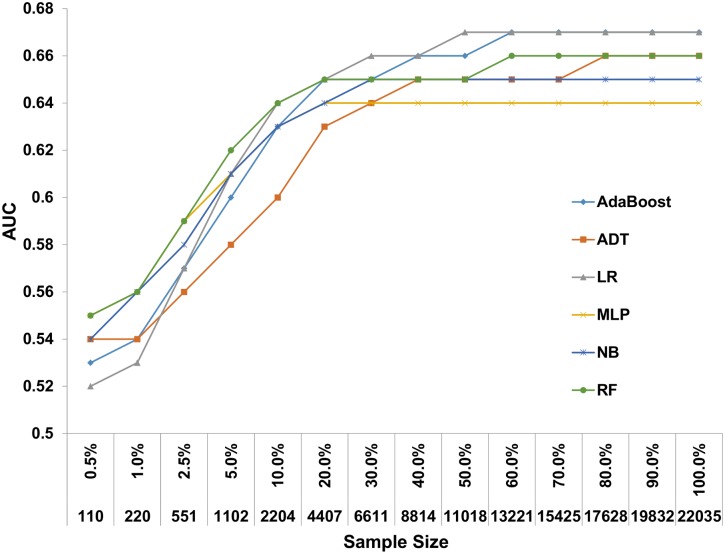
Predictive performance of day 100 NRM prediction models with increasing sample size. A gradually increasing sample from the experimental dataset was introduced to 6 machine learning algorithms. Prediction models were developed for each incremental step and their discriminative performance is plotted on the Y axis. Alternating decision tree (ADT), Logistic regression (LR), Multilayer perceptron (MLP), Naïve base (NB), Random forest (RF).

Day 100 NRM prediction models were developed with 6 ML algorithms on an expanding patient population (110–22,035 patients) sampled from the experimental dataset. When models were developed on all available patients, AUCs ranged from 0.64 for the MLP algorithm to 0.67 for the LR and AdaBoost algorithms (Fig 2 and Table C in S1 File). Depending on the algorithm, predictive performance plateaued on a sample size of 6,611–8,814 patients. Samples consisting of 551 patients or less, demonstrated poor performance with AUCs ranging from 0.56–0.59.

### Sub-population effect on prediction

Prediction models were developed for specific subsets of patients, and performance was compared with models developed on the whole population (Table 2). Performance was significantly lower for the various disease stage subpopulations (e.g., for CR2 AUC ranged from 0.55–0.59) and for patients transplanted from HLA matched unrelated donors. Prediction models developed on the remaining subpopulations had similar performance to models developed using all available patients.

**Table 2:** Predictive performance of day 100 NRM prediction models on varying subpopulations.

		AdaBoost		ADT		LR		MLP		NB		RF		Average performance	
	Sample Size	AUC	STDV	AUC	STDV	AUC	STDV	AUC	STDV	AUC	STDV	AUC	STDV	AUC	STDV
Full dataset	22035	0.67	0.02	0.66	0.02	0.67	0.02	0.63	0.01	0.65	0.02	0.66	0.02	0.66	0.01
Age<45	10820	0.66	0.03	0.65	0.03	0.66	0.03	0.64	0.02	0.65	0.03	0.66	0.03	0.65	0.01
Age> = 45	11215	0.66	0.03	0.65	0.03	0.66	0.03	0.64	0.02	0.65	0.03	0.65	0.03	0.65	0.01
ALL	6214	0.65	0.04	0.64	0.04	0.66	0.04	0.64	0.02	0.64	0.03	0.65	0.03	0.65	0.01
AML	15821	0.67	0.02	0.66	0.03	0.67	0.02	0.65	0.02	0.65	0.02	0.66	0.02	0.66	0.01
CR1	13787	0.63	0.03*	0.61	0.03*	0.64	0.03*	0.63	0.02	0.61	0.03*	0.62	0.03*	0.62	0.01
CR2	4165	0.58	0.05*	0.55	0.04*	0.59	0.05*	0.59	0.03*	0.58	0.05*	0.58	0.05*	0.58	0.01
Advanced	4083	0.62	0.04*	0.61	0.04v	0.61	0.04v	0.59	0.03v	0.6	0.03*	0.61	0.04*	0.61	0.01
MAC	14754	0.66	0.02	0.65	0.02	0.66	0.02	0.63	0.02	0.65	0.02	0.66	0.02	0.65	0.01
RIC	7703	0.67	0.03	0.66	0.03	0.67	0.03	0.66	0.02	0.65	0.03	0.66	0.03	0.66	0.01
MRD	10458	0.65	0.03	0.64	0.03	0.66	0.03	0.65	0.03	0.65	0.03	0.65	0.03	0.65	0.01
MUD	11577	0.64	0.02*	0.63	0.03*	0.64	0.03*	0.62	0.02	0.62	0.03*	0.63	0.02*	0.63	0.01

* p-value <0.05 (t-test), Performance of reach model was compared with the performance of the model developed on the full experimental dataset, with the designated algorithm. Standard deviation (STDV), Alternating decision tree (ADT), Logistic regression (LR), Multilayer perceptron (MLP), Naïve base (NB), Random forest (RF), HLA matched related donor (MRD), HLA matched unrelated donor (MUD).

### Variable importance

**Fig 3:**
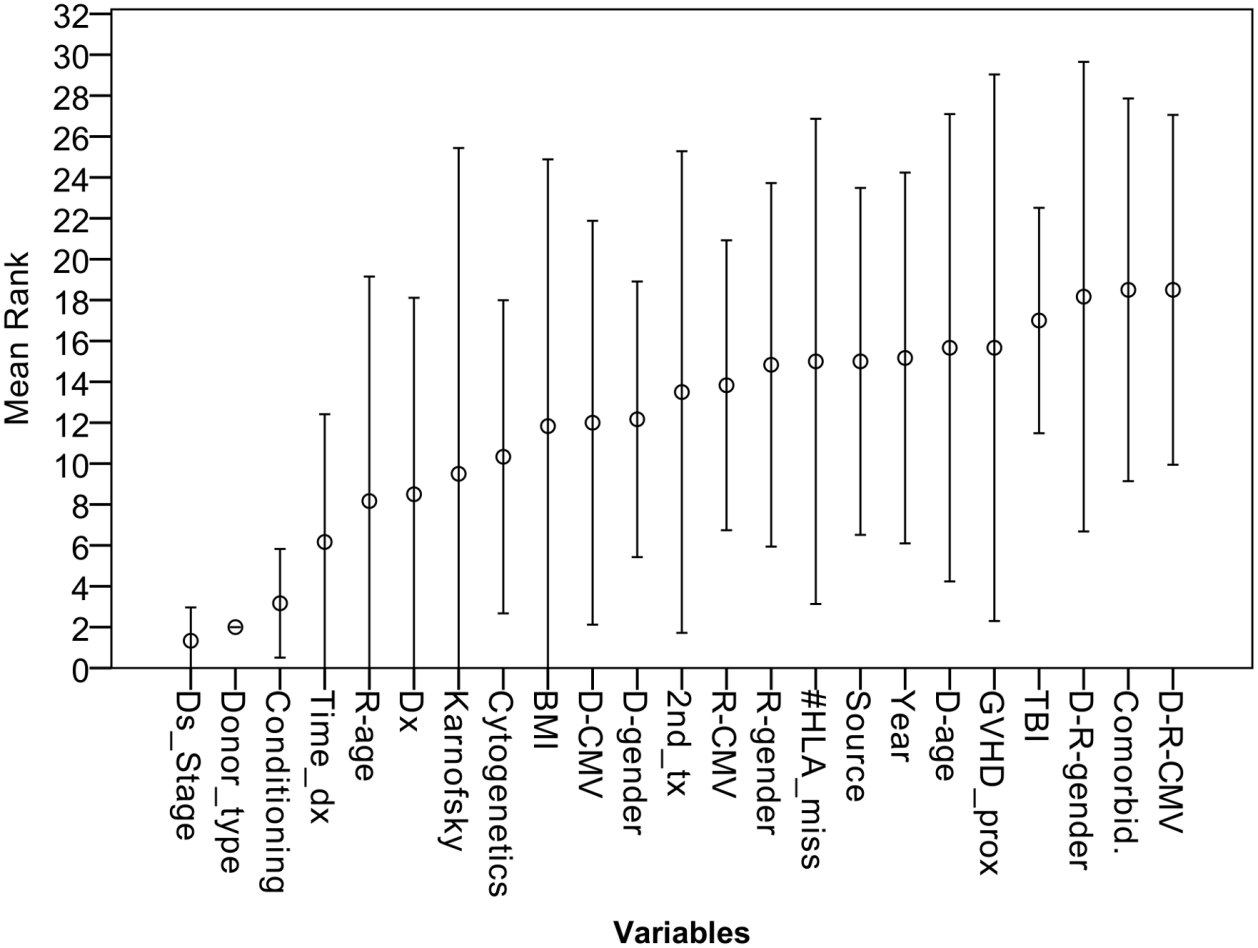
Mean variable ranking of day 100 NRM prediction models. Variable importance were extracted using a feature selection algorithm for 6 machine learning prediction models of day 100 NRM. The circle marks the mean ranking of each variable and the bars describe 2 standard deviations. Disease stage (Ds_Stage); Time from transplant to diagnosis (Time_dx); Diagnosis (Dx); Body mass index (BMI); Donor (D); Recipient (R); Previos autograft (2nd_tx); # of HLA mismatches (#HLA_miss); Graft versus host disease prophylaxis (GVHD_prox); Total body irradiation (TBI);

When the feature selection process was applied on the optimization set, disease stage, donor type and conditioning were consistently the 3 top ranking variables across all day 100 NRM prediction models (Fig 3). The mean variable rankings of time from diagnosis to transplant, recipient age, and diagnosis were 4–6, respectively. However, standard deviation was considerably high, as their importance varied between algorithms.

**Fig 4:**
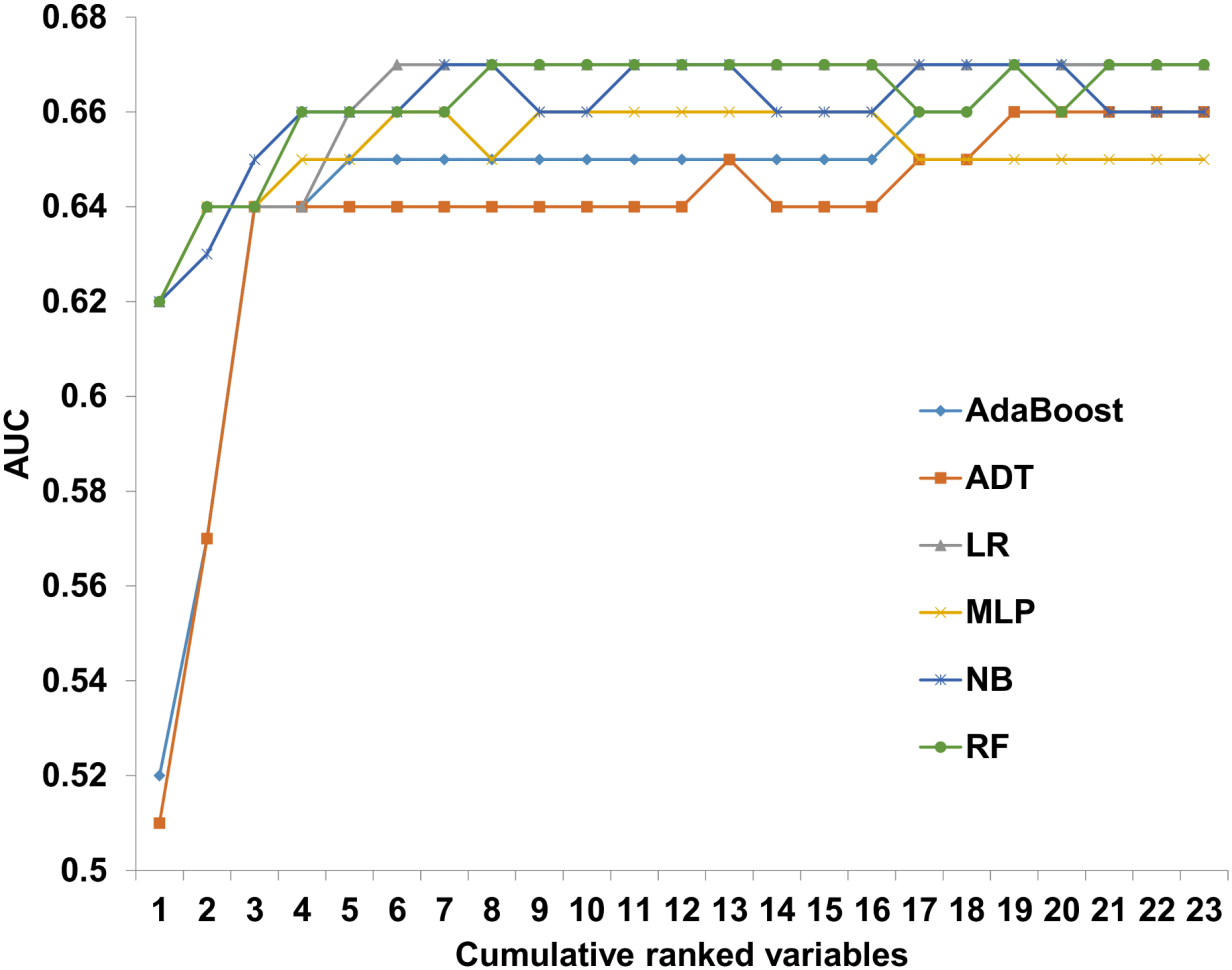
Predictive performance of day 100 NRM prediction models on a cumulative ranked variable list. Alternating decision tree (ADT); Logistic regression (LR); Multilayer perceptron (MLP); Naïve base (NB); Random forest (RF);

To assess the relationship between models' performance and the number of variables incorporated into them, the ranked variables were serially introduced to the 6 ML algorithms. The algorithms were applied on the experimental dataset. Starting from the top ranking variables, gradually adding variables with lower ranking, prediction models for day 100 NRM were iteratively constructed (Fig 1). The maximal predictive performance ranged from 0.65–0.67, with LR and MLP achieving their optimal AUC with only 6 variables (Fig 4). When introduced with the 3 top ranking variables all models achieved an AUC of 0.64.

## Discussion

Eligibility of patients with acute leukemia for allogeneic HSCT is based on a risk benefit-assessment of the relapse risk versus NRM risk [24]. Risk scores for transplant associated mortality have been developed based on retrospective registry data. A large HSCT registry was explored, while automatizing the prediction model development processes, creating thousands of models, depending on the questions asked. We show that for day 100 NRM prediction various models, developed on the basis of 6 popular ML algorithms, reach approximately the same performance. With data commonly collected, saturation of predictive performance requires very few variables, but large datasets.

The nature of association between predictors and response, the data’s quality and dimensionality (i.e., number of variables analyzed), and the number of events per outcome, all affect the sample size necessary for generation of a robust and generalizable prediction model [15]. Hence, predetermination of the sample size is a matter of empirical testing, rather than a standardized calculation. Using repetitive computerized simulations, we demonstrate that with marginal differences between algorithms, approximately 6,000 patients were needed to achieve maximal or near-maximal predictive performance. Defining a strict cutoff for modeling studies would be erroneous, as the data’s features differ between cohorts. However, a rather solid assumption based on the presented results on “real world” data, would be the need to include thousands of patients when aiming to develop and validate similar modeling problems.

Iterative development of prediction models for specific subpopulation has drawn attention to the different disease stage groups. Performance was lowest for the CR2 group, with an AUC ranging from 0.53–0.58. Low performance, but to lesser extent, was also noted for the other disease stage groups. Disease stage is highly predictive of day 100 NRM. Thus, it is not surprising that when disease stage was excluded from the pool of variables considered for prediction, performance declined, as it is highly informative.

Prospects for cure are higher for patients in CR1 compared to other disease stages. Hence, estimation of NRM risk is of special interest in this group, as non-transplant alternatives exist [25–27]. Versluis et al., have addressed such a population receiving reduced intensity conditioning. When looked upon separately, the Hematopoietic Cell Transplantation-Specific Comorbidity Index (HCT-CI) and EBMT score were not predictive of NRM, corroborating the challenge we encountered. A new score, based on integrated feature of the comorbidity index and EBMT score, was constructed achieving an AUC of 0.68 [8].

It should be noted that most algorithms reached an AUC of 0.65 using only 3–5 variables. Adding more variables led to a modest improvement, which translates to marginal clinical significance. The top 3 ranking variables, shared by all algorithms were disease stage, donor type, and conditioning. Transplanters will not be surprised by these determinants, which have been validated repeatedly [28, 29]. Predictive weight attributed to other features varied considerably between models, leading at best to modest increment in predictive accuracy. Traditional HSCT prognostic studies, rely on a collection of variables similar to the one presented. Thus, effective prediction of individualized NRM is unlikely to substantially improve. Incorporation of the HCT-CI score holds promise. However, even when applied separately or in combination with other features, the comorbidity index reaches a maximal AUC of 0.7 [4, 7, 8, 30–32]. In other words, contemporary prognostic models are suitable for risk stratification rather than outcome prediction. The discovery of additional prognostic markers, the incorporation of electronic medical records to routine clinical use, and the addition of biological and genetic data to information gathered on leukemia patients, offers great opportunities for model improvement [33, 34].

Mortality following transplantation is likely the result of a complex network of interactions and non-linear associations. Hence, the Occam's razor concept, where the simplest solution is the best solution, might not hold for prediction of transplantation outcomes. Exploiting the abundance of data now available on transplant patients, could potentially improve prediction models’ applicability. Novel modeling techniques such as ML [35, 36], enabling non-parsimonious incorporation of a high number of variables, are warranted. These methods could potentially improve accuracy, but interpretability might be lost.

The EBMT-ADT prediction model marked the entrance of the data mining methodology into HSCT prognostic research [9, 22]. The aim of the ADT study was development of a prediction model for overall mortality at 100 days following allogeneic HSCT in acute leukemia patients. Though using a data mining methodology, the perspective of the current study was not prediction *per-se*, but rather an analysis of the predictive modeling process and its boundaries, while focusing on NRM at day 100 as the objective. Thousands of prediction models, with varying algorithms, were developed and evaluated in order to discover elements that could improve future models. The *in-silico* experimental system allowed us to dissect the conditions under which the models were developed and the corresponding performance. Thus, providing methodological and clinical insights regarding sample size, modeling technique, and variable importance.

The study carries several limitations. First, it is a retrospective analysis susceptible to data selection and measurement biases. However, the registry analyzed reflects real world data, hence conveying contemporary practice. Second, a few variables suffered from a large amount of missing values. That being said, ML algorithms allow prediction of the outcome of interest without strong assumptions regarding the distribution and missingness, In addition, we show that when discarding variables with more than 15% missing values, prediction does not improve (Table D in S1 File). Third, we focus on short term data- day 100 NRM, rather than long term mortality. We believe that the high rate of day 100 NRM (9.2%) makes it a valid objective. Moreover, prediction of long term outcomes might be expected to give lower performance, as more parameters come into play. Hence, the concepts presented should be applicable to modeling distant outcomes. Fourth, we relate to prediction of day 100 NRM as a simple classification task, disregarding the time to event effect. However, given the large sample size, disregarding censored data (1.3%) is unlikely to have impact on performance.

## Conclusion

The *in-silico* approach is a novel experimental system utilizing machine learning algorithms, for empirical estimation of prediction boundaries in HSCT. Several clinical and methodological lessons have been learned by the suggested approach. Large registry studies, involving thousands of patients are necessary for development of robust prediction models, as performance of different algorithms converged when sampling more than 6,000 patients. In addition, an exhaustive search for variable importance, reveal that few variables "carry the weight" with regard to predictive influence. Potential bias of the presented approach include: data quality issues and selection of a short term rather than a long term outcome. Overall, it appears that when using traditional HSCT data, a point of predictive saturation has been reached. Improving performance will likely require additional types of input like genetic, clonal and biologic factors.

## Supporting Information

S1 File Appendix A in S1 File: Variables’ Definitions. Appendix B in S1 File: Machine Learning Algorithms. Appendix C in S1 File: Feature Selection. Table A in S1 File: Algorithms' parameters. Table B in S1 File: Comparison between variables in the optimization and experimental datasets. Table C in S1 File: Predictive performance of day 100 NRM prediction models with increasing sample size. Table D in S1 File: Predictive performance of day 100 NRM prediction models discarding variables with prevalent missing values.
(DOCX)

## References

[1] Copelan EA. Hematopoietic stem-cell transplantation. The New England journal of medicine. 2006;354(17):1813–26. . 16641398 10.1056/NEJMra052638

[2] Gratwohl A, Hermans J, Goldman JM, Arcese W, Carreras E, Devergie A, et al Risk assessment for patients with chronic myeloid leukaemia before allogeneic blood or marrow transplantation. Chronic Leukemia Working Party of the European Group for Blood and Marrow Transplantation. Lancet. 1998;352(9134):1087–92. . 9798583 10.1016/s0140-6736(98)03030-x

[3] Gratwohl A, Stern M, Brand R, Apperley J, Baldomero H, de Witte T, et al Risk Score for Outcome After Allogeneic Hematopoietic Stem Cell Transplantation A Retrospective Analysis. Cancer. 2009;115(20):4715–26. 10.1002/cncr.24531 WOS:000270740900011. 19642176

[4] Barba P, Martino R, Perez-Simon JA, Fernandez-Aviles F, Castillo N, Pinana JL, et al Combination of the Hematopoietic Cell Transplantation Comorbidity Index and the European Group for Blood and Marrow Transplantation score allows a better stratification of high-risk patients undergoing reduced-toxicity allogeneic hematopoietic cell transplantation. Biol Blood Marrow Transplant. 2014;20(1):66–72. 10.1016/j.bbmt.2013.10.011 . 24141006

[5] Nakaya A, Mori T, Tanaka M, Tomita N, Nakaseko C, Yano S, et al Does the Hematopoietic Cell Transplantation Specific Comorbidity Index (HCT-CI) Predict Transplantation Outcomes? A Prospective Multicenter Validation Study of the Kanto Study Group for Cell Therapy. Biol Blood Marrow Transplant. 2014;20(10):1553–9. 10.1016/j.bbmt.2014.06.005 . 25034961

[6] Parimon T, Au DH, Martin PJ, Chien JW. A risk score for mortality after allogeneic hematopoietic cell transplantation. Annals of internal medicine. 2006;144(6):407–14. . 16549853 10.7326/0003-4819-144-6-200603210-00007

[7] Raimondi R, Tosetto A, Oneto R, Cavazzina R, Rodeghiero F, Bacigalupo A, et al Validation of the Hematopoietic Cell Transplantation-Specific Comorbidity Index: a prospective, multicenter GITMO study. Blood. 2012;120(6):1327–33. 10.1182/blood-2012-03-414573 22740454

[8] Versluis J, Labopin M, Niederwieser D, Socie G, Schlenk RF, Milpied N, et al Prediction of non-relapse mortality in recipients of reduced intensity conditioning allogeneic stem cell transplantation with AML in first complete remission. Leukemia. 2015;29(1):51–7. 10.1038/leu.2014.164 WOS:000347673700006. 24913728

[9] Shouval R, Labopin M, Bondi O, Mishan-Shamay H, Shimoni A, Ciceri F, et al Prediction of Allogeneic Hematopoietic Stem-Cell Transplantation Mortality 100 Days After Transplantation Using a Machine Learning Algorithm: A European Group for Blood and Marrow Transplantation Acute Leukemia Working Party Retrospective Data Mining Study. Journal of Clinical Oncology. 2015:JCO. 2014.59. 1339. 10.1200/JCO.2014.59.1339 26240227

[10] Breiman L. Statistical modeling: The two cultures (with comments and a rejoinder by the author). Statistical Science. 2001;16(3):199–231.

[11] Shouval R, Bondi O, Mishan H, Shimoni A, Unger R, Nagler A. Application of machine learning algorithms for clinical predictive modeling: a data-mining approach in SCT. Bone marrow transplantation. 2014;49(3):332–7. 10.1038/bmt.2013.146 24096823

[12] Shouval R, Nagler A, Labopin M, Unger R. Interpretable Boosted Decision Trees for Prediction of Mortality Following Allogeneic Hematopoietic Stem Cell Transplantation. J Data Mining Genomics Proteomics. 2015;6(4):2.

[13] /MED-AB Forms Manual: http://www.ebmt.org/Contents/Data-Management/Registrystructure/MED-ABdatacollectionforms/Documents/MED-ABFormsManual.pdf.

[14] Collins GS, Reitsma JB, Altman DG, Moons KG. Transparent reporting of a multivariable prediction model for individual prognosis or diagnosis (TRIPOD): the TRIPOD Statement. BMC medicine. 2015;13(1):1. 25563062 10.1186/s12916-014-0241-z PMC4284921

[15] Moons KG, Altman DG, Reitsma JB, Ioannidis JP, Macaskill P, Steyerberg EW, et al Transparent Reporting of a multivariable prediction model for Individual Prognosis Or Diagnosis (TRIPOD): explanation and elaboration. Annals of internal medicine. 2015;162(1):W1–W73. 10.7326/M14-0698 25560730

[16] Breiman L. Random Forests. Machine Learning. 2001;45(1):5–32.

[17] Freund Y, Mason L, editors. The alternating decision tree learning algorithm. ICML; 1999.

[18] Freund Y, Schapire R, Abe N. A short introduction to boosting. Journal-Japanese Society For Artificial Intelligence. 1999;14(771–780):1612.

[19] Han J, Kamber M, Pei J. Data Mining: Concepts and Techniques. 3rd ed: Morgan Kaufmann; 2012.

[20] Hastie T, Tibshirani R, Friedman J, Hastie T, Friedman J, Tibshirani R. The elements of statistical learning: Springer; 2009.

[21] Krogh A. What are artificial neural networks? Nature biotechnology. 2008;26(2):195–7. 10.1038/nbt1386 18259176

[22] Shouval R, Bondi O, Mishan H, Shimoni A, Unger R, Nagler A. Application of machine learning algorithms for clinical predictive modeling: a data-mining approach in SCT. Bone marrow transplantation. 2014;49(3):332–7. 10.1038/bmt.2013.146 . 24096823

[23] Hall MA, Holmes G. Benchmarking attribute selection techniques for discrete class data mining. Knowledge and Data Engineering, IEEE Transactions on. 2003;15(6):1437–47.

[24] Cornelissen JJ, Gratwohl A, Schlenk RF, Sierra J, Bornhauser M, Juliusson G, et al The European LeukemiaNet AML Working Party consensus statement on allogeneic HSCT for patients with AML in remission: an integrated-risk adapted approach. Nature reviews Clinical oncology. 2012;9(10):579–90. 10.1038/nrclinonc.2012.150 . 22949046

[25] Cornelissen JJ, van Putten WL, Verdonck LF, Theobald M, Jacky E, Daenen SM, et al Results of a HOVON/SAKK donor versus no-donor analysis of myeloablative HLA-identical sibling stem cell transplantation in first remission acute myeloid leukemia in young and middle-aged adults: benefits for whom? Blood. 2007;109(9):3658–66. 10.1182/blood-2006-06-025627 . 17213292

[26] Koreth J, Schlenk R, Kopecky KJ, Honda S, Sierra J, Djulbegovic BJ, et al Allogeneic stem cell transplantation for acute myeloid leukemia in first complete remission: systematic review and meta-analysis of prospective clinical trials. Jama. 2009;301(22):2349–61. 10.1001/jama.2009.813 19509382 PMC3163846

[27] Yanada M, Matsuo K, Emi N, Naoe T. Efficacy of allogeneic hematopoietic stem cell transplantation depends on cytogenetic risk for acute myeloid leukemia in first disease remission: a metaanalysis. Cancer. 2005;103(8):1652–8. 10.1002/cncr.20945 . 15742336

[28] Socié G, Stone JV, Wingard JR, Weisdorf D, Henslee-Downey PJ, Bredeson C, et al Long-term survival and late deaths after allogeneic bone marrow transplantation. New England Journal of Medicine. 1999;341(1):14–21. 10387937 10.1056/NEJM199907013410103

[29] Gratwohl A, Stern M, Brand R, Apperley J, Baldomero H, de Witte T, et al Risk score for outcome after allogeneic hematopoietic stem cell transplantation: a retrospective analysis. Cancer. 2009;115(20):4715–26. 10.1002/cncr.24531 19642176

[30] Sorror ML, Giralt S, Sandmaier BM, De Lima M, Shahjahan M, Maloney DG, et al Hematopoietic cell transplantation specific comorbidity index as an outcome predictor for patients with acute myeloid leukemia in first remission: combined FHCRC and MDACC experiences. Blood. 2007;110(13):4606–13. 10.1182/blood-2007-06-096966 17873123 PMC2234788

[31] Teixeira GM, Bittencourt H, de Macedo AV, Martinho GH, Colosimo EA, Rezende SM. Assessing the Influence of Different Comorbidities Indexes on the Outcomes of Allogeneic Hematopoietic Stem Cell Transplantation in a Developing Country. PloS one. 2015;10(9):e0137390 10.1371/journal.pone.0137390 . 26394228 PMC4578937

[32] Sorror ML, Storb RF, Sandmaier BM, Maziarz RT, Pulsipher MA, Maris MB, et al Comorbidity-age index: a clinical measure of biologic age before allogeneic hematopoietic cell transplantation. J Clin Oncol. 2014;32(29):3249–56. 10.1200/JCO.2013.53.8157 25154831 PMC4178523

[33] Dohner H, Weisdorf DJ, Bloomfield CD. Acute Myeloid Leukemia. The New England journal of medicine. 2015;373(12):1136–52. 10.1056/NEJMra1406184 . 26376137

[34] Lauer MS, D'Agostino RB Sr., The randomized registry trial—the next disruptive technology in clinical research? The New England journal of medicine. 2013;369(17):1579–81. 10.1056/NEJMp1310102 23991657

[35] Ishwaran H, Kogalur UB, Blackstone EH, Lauer MS. Random survival forests. The Annals of Applied Statistics. 2008:841–60.

[36] Hsich E, Gorodeski EZ, Blackstone EH, Ishwaran H, Lauer MS. Identifying important risk factors for survival in patient with systolic heart failure using random survival forests. Circulation: Cardiovascular Quality and Outcomes. 2011;4(1):39–45. 21098782 10.1161/CIRCOUTCOMES.110.939371 PMC3991475

