# Supplementary material for: Prediction of Hematopoietic Stem Cell Transplantation Related Mortality- Lessons Learned from the In-Silico Approach: A European Society for Blood and Marrow Transplantation Acute Leukemia Working Party Data Mining Study
Source: PLoS One. 2016 Mar 4;11(3):e0150637. doi: 10.1371/journal.pone.0150637 (PMC4778768; doi:10.1371/journal.pone.0150637)
Supplement: S1 File — Appendix A in S1 File: Variables’ Definitions. Appendix B in S1 File: Machine Learning Algorithms. Appendix C in S1 File: Feature Selection. Table A in S1 File: Algorithms' parameters. Table B in S1 File: Comparison between variables in the optimization and experimental datasets. Table C in S1 File: Predictive performance of day 100 NRM prediction models with increasing sample size. Table D in S1 File: Predictive performance of day 100 NRM prediction models discarding variables with prevalent missing values. (DOCX) [file pone.0150637.s001.docx]

**S1 File**

**Prediction of hematopoietic stem cell transplantation related mortality- lessons learned from the *in-silico* approach: a European society for blood and marrow transplantation acute leukemia working party data mining study**

Shouval R. et al.,

**Appendix A: Variables’ Definitions**

Variables considered include: year of transplant, recipient age, recipient body mass index (BMI), days from diagnosis to transplant, donor age, recipient's and donor's gender and CMV serostatus with possible combinations, Karnofsky score at time of transplant (<80, >=80), diagnosis ((Acute Myeloid Leukemia (AML) or Acute Lymphoblastic Leukemia (ALL)), cytogenetics (poor versus non-poor([1](#_ENREF_1), [2](#_ENREF_2))), disease stage (first/second complete remission (CR), advanced disease), previous autologous HSCTs (no, yes), donor type (sibling or unrelated HLA matched donor), HLA match degree (10/10, 9/10, <9/10), conditioning regimens as defined by Bacigalupo et al. (myeloablative conditioning (MAC), reduced intensity conditioning (RIC)),([3](#_ENREF_3)) Total body irradiation (TBI) as part of the conditioning regimen (no, yes), Graft versus host disease (GVHD) prophylaxis regimens (ex-vivo T cell depletion versus in-vivo T cell depletion versus no T cell depletion).

**Appendix B: Machine Learning Algorithms**

*Naïve bayse* is based on Bayes’ theorem of posterior probability which assumes class conditional independence, meaning that the effect of an attribute value on a given class is independent of the values of the other attributes. Despite its relative simplicity, it is considered a powerful classifier which can predict class membership probabilities.([4](#_ENREF_4))

*AdaBoost* is short for adaptive boosting. The algorithm repeatedly constructs weak models on the training set and adds them to an ensemble, where a weighting vector is adjusted to focus on examples that were misclassified in previous round. The final model is a result of the combined weak models.([5](#_ENREF_5))

*Alternating decision tree* is a combination of decision trees and boosting (i.e., generation of many “weak” models which produces a powerful “committee" for classification), yielding interpretable classification rules with confidence measures.([6](#_ENREF_6))

*Random forest* is an ensemble classiﬁcation technique which sets a consensus prediction for each observation by averaging the results of many individual recursive partitioning tree models. Each of the individual trees is ﬁtted to a randomly selected subset of the observations and uses a random subset of the available predictors at each node as candidates for splitting. Random forests produce robust models, generally avoiding over-fitting.([7](#_ENREF_7))

*Multilayer perceptron* belongs to a class of artificial neural networks (ANNs) algorithms, which are inspired by neuronal learning. One can consider a neuron as a computational unit that receives weighted inputs from others neurons through dendrites, processes them and if a certain threshold is reached, an output is delivered through axon. ANNs are a collection of model neurons interconnected to increase computation/ prediction power. Similar to neurons, connections between units are assigned different weights that are adjusted during training. The input nodes observed are variables used for prediction. The output nodes are the possible outcomes which the network predicts and in between there are accessory computational nodes referred to as hidden layers.([8](#_ENREF_8))

*Logistic regression* is a generalized form of linear regression. It is mainly used for predicting the binary or multi-class dependent variables. As the response variable is discrete, it cannot be modeled directly by linear regression. Therefore, rather than predicting point estimate of the event itself, it builds the model to predict the odds of the occurrence. In a two-class problem, odds greater than 50% would mean that the case is assigned to the class designated as ‘‘1’’ and ‘‘0’’ otherwise. While logistic regression is a very powerful modeling tool, it assumes that the response variable (the log odds, not the event itself) is linear with respect to the predictor variables.([9](#_ENREF_9))

**Appendix C: Feature Selection**

Using a classifier based feature selection algorithm (classifierBasedAttributeSelection package, WEKA v.3-7-11, Table S1), which was applied on the optimization dataset for each of the 6 previously described ML classification algorithms, variables were ranked according to their importance for prediction of day 100 NRM. Importance is estimated by removing an evaluated variable from the dataset and running a classifier (i.e., a selected ML classification algorithm) with 10 fold cross-validation on the reduced dataset, generating a performance estimate for this variable which is calculated by subtracting the performance estimate without the variable from the performance achieved with the full dataset including this variable. Thus, redundancies are avoided and the extent of individual variable contribution to overall performance, on top of all other available variables, is determined.

**Table A: Algorithms' parameters**

| **Algorithm** | **Tunded algorithms' parameters** | **Feature selection algorithm parameters** |
| --- | --- | --- |
| AdaBoost | weka.classifiers.meta.AdaBoostM1 -- -P 100 -S 1 -I 100 -Wweka.classifiers.trees.DecisionStump | weka.experiment.CrossValidationResultProducer -X 10 -O splitEvalutorOut.zip -W weka.experiment.ClassifierSplitEvaluator -- -W weka.classifiers.meta.AttributeSelectedClassifier -I 0 -C 1 -- -E "weka.attributeSelection.ClassifierAttributeEval -execution-slots 1 -B weka.classifiers.meta.AdaBoostM1 -F 5 -T 0.01 -R 1 -E auc -- -P 100 -S 1 -I 100 -W weka.classifiers.trees.DecisionStump" -S "weka.attributeSelection.Ranker -T -1.7976931348623157E308 -N 23" -W weka.classifiers.meta.AdaBoostM1 -- -P 100 -S 1 -I 100 -W weka.classifiers.trees.DecisionStump |
| Alternating Decision Trees | weka.classifiers.trees.ADTree -- -B 10 -E -3 -S 1 | weka.experiment.CrossValidationResultProducer -X 10 -O splitEvalutorOut.zip -W weka.experiment.ClassifierSplitEvaluator -- -W weka.classifiers.meta.AttributeSelectedClassifier -I 0 -C 1 -- -E "weka.attributeSelection.ClassifierAttributeEval -execution-slots 1 -B weka.classifiers.trees.ADTree -F 5 -T 0.01 -R 1 -E auc -- -B 10 -E -3 -S 1" -S "weka.attributeSelection.Ranker -T -1.7976931348623157E308 -N 23" -W weka.classifiers.trees.ADTree -- -B 10 -E -3 -S 1 |
| Logistic Regression | weka.classifiers.functions.Logistic -- -R 1.0E-8 -M -1 | weka.experiment.CrossValidationResultProducer -X 10 -O splitEvalutorOut.zip -W weka.experiment.ClassifierSplitEvaluator -- -W weka.classifiers.meta.AttributeSelectedClassifier -I 0 -C 1 -- -E "weka.attributeSelection.ClassifierAttributeEval -execution-slots 1 -B weka.classifiers.functions.Logistic -F 5 -T 0.01 -R 1 -E auc -- -R 1.0E-8 -M -1" -S "weka.attributeSelection.Ranker -T -1.7976931348623157E308 -N 23" -W weka.classifiers.functions.Logistic -- -R 1.0E-8 -M -1 |
| Multi-Layer Perceptron | MultilayerPerceptron -- -L 0.5 -M 0.4 -N 500 -V 0 -S 0 -E 20 -H a -D | weka.experiment.CrossValidationResultProducer -X 10 -O splitEvalutorOut.zip -W weka.experiment.ClassifierSplitEvaluator -- -W weka.classifiers.meta.AttributeSelectedClassifier -I 0 -C 1 -- -E "weka.attributeSelection.ClassifierAttributeEval -execution-slots 1 -B weka.classifiers.functions.MultilayerPerceptron -F 5 -T 0.01 -R 1 -E auc -- -L 0.5 -M 0.4 -N 500 -V 0 -S 0 -E 20 -H a" -S "weka.attributeSelection.Ranker -T -1.7976931348623157E308 -N 23" -W weka.classifiers.functions.MultilayerPerceptron -- -L 0.5 -M 0.4 -N 500 -V 0 -S 0 -E 20 -H a |
| Naïve Bayes | weka.classifiers.bayes.NaiveBayes | weka.experiment.CrossValidationResultProducer -X 10 -O splitEvalutorOut.zip -W weka.experiment.ClassifierSplitEvaluator -- -W weka.classifiers.meta.AttributeSelectedClassifier -I 0 -C 1 -- -E "weka.attributeSelection.ClassifierAttributeEval -execution-slots 1 -B weka.classifiers.bayes.NaiveBayes -F 5 -T 0.01 -R 1 -E auc --" -S "weka.attributeSelection.Ranker -T -1.7976931348623157E308 -N 23" -W weka.classifiers.bayes.NaiveBayes |
| Random Forest | RandomForest -- -I 100 -K 1 -S 1 -depth 3 -num-slots 1 | weka.experiment.CrossValidationResultProducer -X 10 -O splitEvalutorOut.zip -W weka.experiment.ClassifierSplitEvaluator -- -W weka.classifiers.meta.AttributeSelectedClassifier -I 0 -C 1 -- -E "weka.attributeSelection.ClassifierAttributeEval -execution-slots 1 -B weka.classifiers.trees.RandomForest -F 5 -T 0.01 -R 1 -E auc -- -I 100 -K 1 -S 1 -depth 3 -num-slots 1" -S "weka.attributeSelection.Ranker -T -1.7976931348623157E308 -N 23" -W weka.classifiers.trees.RandomForest -- -I 100 -K 1 -S 1 -depth 3 -num-slots 1 |

**Table B: Comparison between variables in the optimization and experimental datasets**

|  |  | Optimization dataset | | Analysis dataset | |
| --- | --- | --- | --- | --- | --- |
|  |  | Value | N |  | N |
| Mean year (SD) |  | 2009 (3) | 3888 | 2009 (3) | 22035 |
| Mean recipient age (SD) |  | 44 (14) | 3888 | 44 (14) | 22035 |
| Mean BMI (SD) |  | 25 (5) | 1408 | 25 (5) | 7942 |
| Mean days between diagnosis and HSCT (SD) |  | 370 (568) | 3888 | 365 (556) | 22035 |
| Mean donor's age (SD) |  | 39 (13) | 1483 | 39 (13) | 8544 |
|  | Value | N | % | N | % |
| Recipient gender | Male | 2108 | 54.3% | 12120 | 55.1% |
|  | Female | 1771 | 45.7% | 9873 | 44.9% |
| Recipient CMV serostatus | - | 1147 | 33.3% | 6641 | 34.2% |
|  | + | 2298 | 66.7% | 12769 | 65.8% |
| Karnofsky at transplant | >=80 | 3413 | 94.0% | 19553 | 94.3% |
|  | <80 | 217 | 6.0% | 1186 | 5.7% |
| Diagnosis | AML | 2789 | 71.7% | 15821 | 71.8% |
|  | ALL | 1099 | 28.3% | 6214 | 28.2% |
| Cytogenetics risk | Standard | 1533 | 75.7% | 8547 | 74.9% |
|  | Poor | 493 | 24.3% | 2857 | 25.1% |
| Disease stage | CR1 | 2414 | 62.1% | 13787 | 62.6% |
|  | CR2 | 744 | 19.1% | 4165 | 18.9% |
|  | Advanced | 730 | 18.8% | 4083 | 18.5% |
| Previous autograft | - | 3792 | 97.5% | 21443 | 97.3% |
|  | + | 96 | 2.5% | 592 | 2.7% |
| Donor gender | Male | 2312 | 61.0% | 13400 | 62.1% |
|  | Female | 1481 | 39.0% | 8164 | 37.9% |
| Donor CMV serostatus | - | 1623 | 47.4% | 9304 | 48.2% |
|  | + | 1803 | 52.6% | 9996 | 51.8% |
| D-R sex combination | Male D to male R | 1337 | 35.3% | 7816 | 36.3% |
|  | Female D to female R | 764 | 20.2% | 4099 | 19.0% |
|  | Male D to female R | 967 | 25.5% | 5561 | 25.8% |
|  | Female D to male R | 717 | 18.9% | 4057 | 18.8% |
| D-R CMV serostatus combination | D-CMV–/R-CMV– | 824 | 24.4% | 4748 | 25.0% |
|  | D-CMV+/R-CMV– or D-CMV–/R-CMV+ | 1316 | 39.0% | 7601 | 40.0% |
|  | D-CMV+/R-CMV+ | 1238 | 36.6% | 6668 | 35.1% |
| Donor type | HLA matched unrelated donor | 2008 | 51.6% | 11577 | 52.5% |
|  | HLA identical sibling | 1880 | 48.4% | 10458 | 47.5% |
| HLA match degree | 10/10 | 942 | 70.5% | 5577 | 71.9% |
|  | 9/10 | 331 | 24.8% | 1737 | 22.4% |
|  | <9/10 | 63 | 4.7% | 440 | 5.7% |
| Source of stem cells | BM | 631 | 16.2% | 3478 | 15.8% |
|  | PB or BM+PB | 3257 | 83.8% | 18557 | 84.2% |
| Conditioning | MAC | 2504 | 65.8% | 14332 | 66.3% |
|  | RIC | 1303 | 34.2% | 7281 | 33.7% |
| TBI | 0 | 2217 | 57.5% | 12825 | 58.6% |
|  | 1 | 1642 | 42.5% | 9058 | 41.4% |
| GVHD prevention | Ex-vivo T cell depletion | 125 | 3.6% | 675 | 3.4% |
|  | In-vivo T cell depletion | 1478 | 42.2% | 8347 | 42.3% |
|  | No T cell depletion | 1896 | 54.2% | 10707 | 54.3% |
| Relapse at day 100 | - | 3507 | 90.2% | 19877 | 90.2% |
|  | + | 381 | 9.8% | 2158 | 9.8% |
| Non relapse related mortality at day 100 | .00 | 3513 | 90.4% | 20023 | 90.9% |
|  | 1.00 | 375 | 9.6% | 2012 | 9.1% |
| Overall mortality at day 100 | .00 | 3378 | 86.9% | 19265 | 87.4% |
|  | 1.00 | 510 | 13.1% | 2770 | 12.6% |

[Interquartile range](http://en.wikipedia.org/wiki/Interquartile_range) (IQR), Hematopoietic stem cell transplantation (HSCT), Body mass index (BMI), Acute myeloid leukemia (AML), Acute lymphoblastic leukemia (ALL), First complete remission (CR1), Second complete remission (CR2), Recipient (R), Donor (D), Cytomegalovirus (CMV), Myeloablative conditioning (MAC), Reduced intensity conditioning (RIC), Total body irradiation (TBI), Graft versus host disease (GVHD), Antithymocyte globulin (ATG), Peripheral blood (PB), Bone marrow (BM)

**Table C**: **Predictive performance of day 100 NRM prediction models with increasing sample size**

| **Dataset Size (n)** | **%** | **AdaBoost** | | **ADT** | | **LR** | | **MLP** | | **NB** | | **RF** | |
| --- | --- | --- | --- | --- | --- | --- | --- | --- | --- | --- | --- | --- | --- |
|  |  | **AUC** | **SD** | **AUC** | **SD** | **AUC** | **SD** | **AUC** | **SD** | **AUC** | **SD** | **AUC** | **SD** |
| 110 | 0.5 | 0.53 | 0.03* | 0.54 | 0.03* | 0.52 | 0.03* | 0.55 | 0.04* | 0.54 | 0.03* | 0.55 | 0.04* |
| 220 | 1 | 0.54 | 0.03* | 0.54 | 0.03* | 0.53 | 0.04* | 0.56 | 0.04* | 0.56 | 0.04* | 0.56 | 0.03* |
| 551 | 2.5 | 0.57 | 0.03* | 0.56 | 0.03* | 0.57 | 0.03* | 0.59 | 0.04* | 0.58 | 0.03* | 0.59 | 0.03* |
| 1102 | 5 | 0.6 | 0.03* | 0.58 | 0.03* | 0.61 | 0.02* | 0.61 | 0.02* | 0.61 | 0.03* | 0.62 | 0.03* |
| 2204 | 10 | 0.63 | 0.02* | 0.6 | 0.02* | 0.64 | 0.02* | 0.63 | 0.02 | 0.63 | 0.02* | 0.64 | 0.02* |
| 4407 | 20 | 0.65 | 0.02* | 0.63 | 0.02* | 0.65 | 0.02* | 0.64 | 0.02 | 0.64 | 0.02* | 0.65 | 0.02* |
| 6611 | 30 | 0.65 | 0.02* | 0.64 | 0.02* | 0.66 | 0.02* | 0.64 | 0.02 | 0.65 | 0.02 | 0.65 | 0.02 |
| 8814 | 40 | 0.66 | 0.02* | 0.65 | 0.02* | 0.66 | 0.02 | 0.64 | 0.02 | 0.65 | 0.02 | 0.65 | 0.02 |
| 11018 | 50 | 0.66 | 0.02 | 0.65 | 0.02 | 0.67 | 0.02 | 0.64 | 0.02 | 0.65 | 0.02 | 0.65 | 0.02 |
| 13221 | 60 | 0.67 | 0.02 | 0.65 | 0.02 | 0.67 | 0.02 | 0.64 | 0.02 | 0.65 | 0.02 | 0.66 | 0.02 |
| 15425 | 70 | 0.67 | 0.02 | 0.65 | 0.02 | 0.67 | 0.02 | 0.64 | 0.02 | 0.65 | 0.02 | 0.66 | 0.02 |
| 17628 | 80 | 0.67 | 0.02 | 0.66 | 0.02 | 0.67 | 0.02 | 0.64 | 0.02 | 0.65 | 0.02 | 0.66 | 0.02 |
| 19832 | 90 | 0.67 | 0.02 | 0.66 | 0.02 | 0.67 | 0.02 | 0.64 | 0.02 | 0.65 | 0.02 | 0.66 | 0.02 |
| 22035 | 100 | 0.67 | 0.02 | 0.66 | 0.02 | 0.67 | 0.02 | 0.64 | 0.02 | 0.65 | 0.02 | 0.66 | 0.02 |

* p-value <0.05 (t-test), Performance of reach model was compared with the performance of the model developed on 100% of the population, with the designated algorithm.

Non relapse mortality (NRM); Area under curve (AUC); Standard deviation (SD); Alternating decision tree (ADT); Logistic regression (LR); Multilayer perceptron (MLP); Naïve base (NB); Random forest (RF); Area under curve (AUC); Standard deviation (SD).

**Table D: Predictive performance of day 100 NRM prediction models discarding variables with prevalent missing values***

| **Algorithm** | **AUC** | **SD** |
| --- | --- | --- |
| AdaBoost | 0.67 | 0.02 |
| ADT | 0.66 | 0.02 |
| LR | 0.67 | 0.02 |
| MLP | 0.64 | 0.02 |
| NB | 0.65 | 0.02 |
| RF | 0.66 | 0.02 |

Non relapse mortality (NRM); Area under curve (AUC); Standard deviation (SD); Alternating decision tree (ADT); Logistic regression (LR); Multilayer perceptron (MLP); Naïve base (NB); Random forest (RF); Area under curve (AUC); Standard deviation (SD).

*Variables with more than 15% missing values were excluded. These include body mass index, cytogenetics, donor's age, and degree of HLA mismatch.

**References**

1. Pui CH, Robison LL, Look AT. Acute lymphoblastic leukaemia. Lancet. 2008 Mar 22;371(9617):1030-43.

2. Dohner H, Estey EH, Amadori S, Appelbaum FR, Buchner T, Burnett AK, et al. Diagnosis and management of acute myeloid leukemia in adults: recommendations from an international expert panel, on behalf of the European LeukemiaNet. Blood. 2010 Jan 21;115(3):453-74.

3. Bacigalupo A, Ballen K, Rizzo D, Giralt S, Lazarus H, Ho V, et al. Defining the intensity of conditioning regimens: working definitions. Biol Blood Marrow Transplant. 2009 Dec;15(12):1628-33.

4. Han J, Kamber M, Pei J. Data Mining: Concepts and Techniques. 3rd ed: Morgan Kaufmann, 2012.

5. Freund Y, Schapire R, Abe N. A short introduction to boosting. Journal-Japanese Society For Artificial Intelligence. 1999;14(771-780):1612.

6. Freund Y, Mason L. The alternating decision tree learning algorithm. ICML; 1999; 1999. p. 124-33.

7. Breiman L. Random Forests. Machine Learning. 2001;45(1):5-32.

8. Krogh A. What are artificial neural networks? Nature biotechnology. 2008 Feb;26(2):195-7.

9. Hastie T, Tibshirani R, Friedman J, Hastie T, Friedman J, Tibshirani R. The elements of statistical learning: Springer, 2009.
